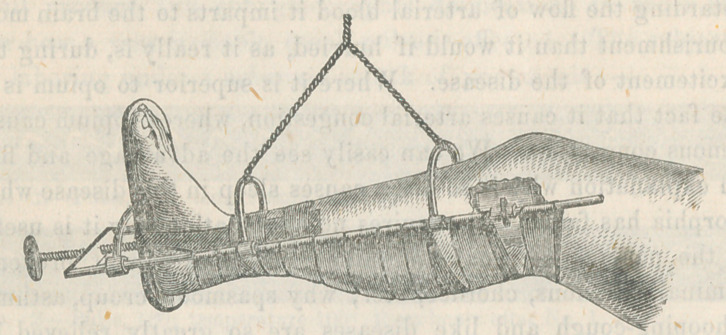# A New Fracture Apparatus

**Published:** 1869-05

**Authors:** Edmund Andrews

**Affiliations:** 81 Monroe St., Chicago


ARTICLE XIX.

### A NEW FRACTURE APPARATUS.

A new dressing for fractures of the leg has been devised by Dr. E. D. Kittoe, of Galena, late medical inspector U. S. A., which, on account of its convenience and capability of being made by an ordinary mechanic, is deserving of commendation. It consists of two steel or iron rods a quarter of an inch in diameter and thirty inches in length. These are connected by a cross-bar at one end and by two sliding semicircles intended to go over the front of the leg. The accompanying engraving will illustrate the form:—

A foot-piece of wood sits between the rods, and is suspended to a steel semicircle by a clamp screw, so as to allow of its being turned in any direction, and also drawn to and fro on the rods, which are loosely clasped by the semicircle. The foot of the patient is attached to the board by adhesive straps either passed around the foot itself, or up the sides of the leg, as shown in the engraving. Through the cross-bar at the end of the rods passes a screw ten inches long, which is attached to the foot-piece by a swivel joint. By these means the foot-piece is drawn down, making extension with any desired force. The counter-extension is made by two broad, thick pads, attached near the upper extremities of the side rods by clamp screws, so that they can be adjusted to any necessary length. These pads press against the bulge of the tibia on each side, just below the knee, and may be fitted to variable sizes by extemporized pads placed under them, thus making effectual counter-extension. The limb is supported between the rods by passing a bandage to and fro beneath it exactly in the manner of Smith's Anterior Splint; and the whole is then suspended by a branched cord to a hook in the ceiling, or to a wire arch under the bed-clothes. The branched cord is of course attached to the two sliding arcs which attach to the rods, as seen in the wood-cut.

The only cases where this splint cannot be used would seem to be those in which there has been contusion about the knee, so that the patient cannot tolerate the pressure of the counter-extension pads; but even in these cases it would have all the advantages of Smith's Anterior Splint, while in all other cases it adds to the merits of Smith's apparatus the benefits of perfect extension and counter-extension. It is a great comfort to a patient to have the free motion of the limb allowed by suspension, without losing the advantage of extension, or risking the displacement of the bones. Both these objects are gained by Dr. Kittoe's apparatus.

EDMUND ANDREWS, M.D.

*81 Monroe St., Chicago.*

## Figures and Tables

**Figure f1:**